# A Family History of Dilated Cardiomyopathy Induced by Viral Myocarditis

**DOI:** 10.1155/2012/204371

**Published:** 2012-02-27

**Authors:** Thomas Cognet, Olivier Lairez, Pauline Marchal, Jérôme Roncalli, Michel Galinier

**Affiliations:** ^1^Department of Cardiology, University Hospital of Rangueil, 1 Avenue Jean Poulhès, 31059 Toulouse Cedex 9, France; ^2^Cardiac Imaging Center, University Hospital of Toulouse, Toulouse, France; ^3^Department of Nuclear Medicine, University Hospital of Toulouse, Toulouse, France; ^4^Rangueil Medical School, Toulouse, France; ^5^Purpan Medical School, Toulouse, France

## Abstract

Myocarditis can lead to acute heart failure, cardiogenic shock, or sudden death and later, dilated cardiomyopathy (DCM) with chronic heart failure. We report the cases of two DCM induced by acute and past myocarditis in the same family and expressed by its two main complications within few weeks: an hemodynamic presentation as a fulminant myocarditis rapidly leading to cardiac tranplantation and a rythmologic presentation as an electrical storm leading to catheter ablation of ventricular tachycardia. These cases ask the question of the family predisposition to viral myocarditis leading to DCM.

## 1. Introduction

A family history of dilated cardiomyopathy (DCM) is found in about one-third to one-half of patients with idiopathic DCM, who are considered to have familial DCM. DCM can also be the consequence of myocarditis, but not every acute myocarditis develops DCM. These findings suggest that there could be familial predisposition to infection in some patients. Familial DCM is commonly reattached to genetic mutations that affect myocardial functions but there is no data to support the possibility of mutation affecting sensibility to infection.

We report two cases of DCM developing in the same family within a few weeks, with confirmed infectious aetiology in one of them.

## 2. Case Presentation

**Figure 1:**
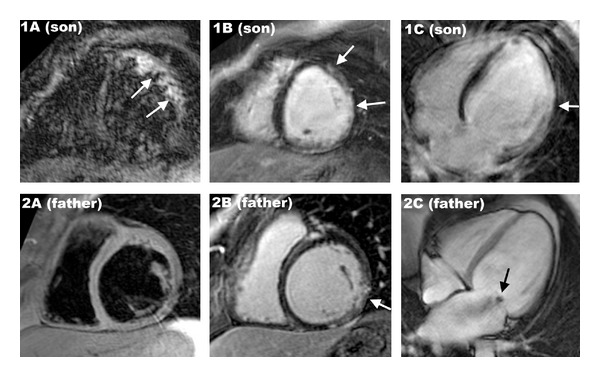
Panel A: son short-axis view T2 weight cardiac MRI with black blood and fat saturation showing bright signal intensity of the lateral wall (white arrows) suggesting myocardial oedema. Panels 1B and 1C: son short-axis view (1B) and 4-chamber view (1C) cardiac MRI showing subepicardial late gadolinium enhancement of the lateral wall (white arrows) suggesting diagnosis of myocarditis. Panel 2A: father short-axis view T2 weight cardiac MRI with black blood and fat saturation without signal abnormality. Panel 2B: father short-axis view (1B) cardiac MRI showing subepicardial late gadolinium enhancement of the inferolateral wall (white arrow) suggesting diagnosis of myocarditis. Panel 2C: father four-chamber view cine-MRI during the first part of the systole showing dilated cardiomyopathy with functional mitral regurgitation (black arrow).

A 14-year-old boy referred for fulminant myocarditis diagnosed by cardiac magnetic resonance imaging (MRI): left ventricle was dilated with an left ventricle ejection fraction (LVEF) of 15% and apical thrombus, lateral wall displays hypersignal in the T2 weight sequences (Figure 1 panel 1A) and subepicardial late gadolinium enhancement (Figure 1 panels 1B and 1C). Electrocardiogram showed sinus rhythm with complete left bundle branch block. Seroconversion to parvovirus B19 was detected in patient's serum and lab tests identified strong inflammation. Myocardial biopsies confirmed the diagnosis of acute myocarditis finding parvovirus B19 and active inflammation within the myocardium. Healthy myocardial tissue was also found excluding previous cardiomyopathy. Rapidly, he presented a refractory cardiogenic shock that needed extracorporeal membrane oxygenation (ECMO) support then cardiac transplantation. Surgery was successful and anatomopathology on native heart confirmed the final diagnostic of fulminant myocarditis induced by Parvovirus B19.

**Figure 2:**
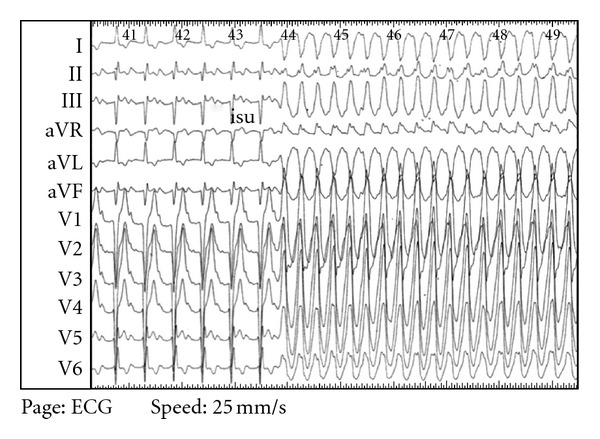
Twelve leads electrocardiogram during endocavitary exploration showing basal-mitral ventricular tachycardia triggered by isoprenaline.

Two months later, his father, a previously healthy 47-year-old man with no cardiovascular history, was referred for an electrical storm. There was no family history of sudden death. The ECG revealed monomorphic sustained ventricular tachycardia with a right side behind right axis. Coronary artery disease was excluded by coronary angiography. Cardiac MRI was performed showing a DC with severe impairment of the LVEF, functional mitral regurgitation (Figure 1 panel 2C), and subepicardial inferoseptal late gadolinium enhancement (Figure 1 panel 2B). There was no signal abnormality in the T2 weight sequences (Figure 1 panel 2A) suggesting an aged myocarditis without active inflammation. Myocardial biopsies found typical pattern of DCM with cardiomyocyte hypertrophy with myofibrillogenesis and vacuolar dystrophy and interstitial and perivascular fibrosis. No germ neither other aetiology (such as bacterial, fungal, hypersensitivity, or immunologic aetiologies) was found. Maximal medical treatment was not efficient to stop the electrical storm. He underwent ablation of a nonreentrant ventricular tachycardia (Figure 2) and cardioverter defibrillator implantation. Clinical evolution was favourable.

## 3. Discussion

This family history of myocarditis underlies a genetic susceptibility. Most of viruses in myocarditis are common cough viruses and the reason why some people develop myocardial damages remains unclear. Studies show that inflammation and viral persistence in the myocardium are associated with progressive impairment of left ventricle function [1]. During the pathophysiological process of viral myocarditis, autoimmune myocardial injury can follow direct-virus-mediated myocardial damage resulting in DCM [2]. The importance of genetic complexity and polymorphisms was confirmed by the study of autoimmune myocarditis [3]. In mice, several genes have been identified conferring susceptibility for viral induced-chronic myocarditis. Some of them belong to major histocompatibility complex, others are close to loci for autoimmune diseases, highlighting the link between genetics and immune response to viral injury and DCM [4]. The link between genetic susceptibility, related immune response, and DC is not proven in human. It could explain why some people develop clinical symptoms of myocarditis and severe outcomes, whereas others remain free from complications.

## 4. Conclusion

Familial DCM may have its origin in myocarditis, and therefore identification of genes implicated and of immunes responses could improve therapies.
